# Integrated Nutrient Management Enhances Productivity and Nitrogen Use Efficiency of Crops in Acidic and Charland Soils

**DOI:** 10.3390/plants10112547

**Published:** 2021-11-22

**Authors:** Mohammad Mojibur Rahman, Shihab Uddin, Mohammad Mofizur Rahman Jahangir, Zakaria M. Solaiman, Saud Alamri, Manzer H. Siddiqui, Mohammad Rafiqul Islam

**Affiliations:** 1 Department of Soil Science, Bangladesh Agricultural University, Mymensingh 2202, Bangladesh; mojiburbari@gmail.com (M.M.R.); shihab43151@bau.edu.bd (S.U.); mmrjahangir@bau.edu.bd (M.M.R.J.); 2 Bangladesh Agricultural Research Institute, Gazipur 1701, Bangladesh; 3 UWA School of Agriculture and Environment, The UWA Institute of Agriculture, The University of Western Australia, Perth, WA 6009, Australia; zakaria.solaiman@uwa.edu.au; 4 Department of Botany and Microbiology, College of Science, King Saud University, Riyadh 2455, Saudi Arabia; saualamri@ksu.edu.sa (S.A.); mhsiddiqui@ksu.edu.sa (M.H.S.)

**Keywords:** biochar, compost, aggregate size distribution, crop productivity, nitrogen uptake, nitrogen use efficiency

## Abstract

Integrated Plant Nutrient System (IPNS) is practiced worldwide to maintain soil quality. Two field experiments were conducted in 2019 and 2020 in acidic and charland soils to assess the impact of different manures, viz., poultry manure (PM), vermicompost (VC), compost (OF), rice husk biochar (RHB), poultry manure biochar (PMB)-based IPNS, and dolomite over control on productivity and nitrogen use efficiency (NUE) of the Mustard-Boro-Transplanted Aman and Maize-Jute-Transplanted Aman cropping patterns, and on soil properties. The experiments were laid out in a randomized complete block design with four replications. The results showed that IPNS treatments significantly improved soil aggregate properties and total nitrogen in acidic soil, and bulk density in charland soil. In both years, IPNS treatments increased system productivity from 55.4 to 82.8% in acidic soil and from 43.3 to 115.4% in charland soil over that of control. IPNS and dolomite treatments increased nitrogen uptake from 35.5 to 105.7% over that of control and NUE in both soils in 2019 and 2020. PMB- and OF-based INPS treatments exhibited superior performances in both soils, and the impact was more prominent in 2020. Therefore, PMB- and OF-based IPNS can be recommended for maximizing system productivity and NUE with concurrent improvement of physicochemical properties of acidic and charland soils.

## 1. Introduction

The increasing growth rate of the world’s population will necessitate a significant increase in agricultural production to meet the demand by 2025 [1], which will increase land degradation due to intensive agricultural use and indiscriminate fertilizer application. Soil physical, chemical, and biological properties like organic carbon, hydraulic conductivity, BD, runoff and erosion, nutrient availability, microbial biomass, and enzyme activities are severely affected by land degradation [2,3,4]. Acidic and charland soils are degraded soils in Bangladesh with low organic matter and nutrient status, threatening crop production of about 36% of land [5,6], as well as 57% of the world’s land coverage [7]. Soil acidity affects plant growth by influencing soil microbes, nutrient leaching, and nutrient and toxic element availability. Charland soil is mainly a major concern in the Bengal Delta of India and Bangladesh for crop production because of its sandy type soil texture, low water holding capacity, and low nutrient status. Thus, these two soils need to be ameliorated to maintain soil health and quality for sustainable crops and increased crop production to ensure food security for the country.

Farmers were using organic manures such as poultry manure (PM), compost (OF), cowdung (CD), and others for their beneficial effects on soil health by improving soil physicochemical properties and by increasing macro- and micronutrient availability since ancient times [8,9]. Biochar, a carbon-rich compound, resulting from the pyrolysis process of different biomasses acts as an alternative or complementary organic amendment [10,11]. Positive effects of biochar application on soil physical, chemical, and biological properties and on crop yield were reported earlier [12,13,14], although no or adverse effects on soil properties and crop yield in sandy loam and Fort Collins loam soils having a pH of 6.15 and 8.70 were also reported [15,16]. The incorporation of biochar derived from rice husk into soils could significantly improve soil physicochemical properties, such as soil moisture content, water holding capacity, BD, available-N nutrients, etc., in paddy fields, [17] and thereby increase crop yield [18,19]. Biochar application decreased soil BD, whereas increased porosity, available soil water content, organic carbon (OC), soil pH, available P, cation exchange capacity (CEC), exchangeable K, and Ca were observed [20]. Biochar produced from animal origins may have higher nutrient content, but its agronomic value as a soil conditioner was not investigated [19]. Several authors reported that vermicompost (VC) amendment acts as a slow-release fertilizer and can directly increase crop production through increased availability of plant nutrients. It indirectly promotes soil quality by improving soil structure and stimulating microbial activity relative to conventional chemical fertilization [21,22,23]. Application of dolomite in combination with PM and chemical fertilizer increases crop productivity, N uptake, and NUE, as well as improves the physicochemical properties of acidic soil [7,24,25].

The Integrated Plant Nutrient System (IPNS) is a combination of organic and inorganic fertilizers that can be used to reduce the use of chemical fertilizers by establishing a balance between fertilizer inputs and crop nutrient requirements, thereby maintaining soil fertility, restoring soil health, and providing plants with nutrient requirements continuously [26,27,28]. Bilkis et al. [29] reported that PM and VC based INPS approaches increased yield and nutrient uptake of the Boro-Fallow-T. Aman cropping pattern as well as soil fertility. Saha et al. [30] observed that the addition of NPK fertilizers and organic manure, lime, and biofertilizers increased soil organic carbon (SOC) content, aggregate stability, moisture-retention capacity, and infiltration rate of the soil while reducing BD. Chaudhry et al. [31] reported that biochar and 50% of recommended dose of NPK was most effective for improving soil physico-chemical properties viz., BD, particle density, porosity, pH, EC, organic matter, SOC, total N, available P, K, soil microbial biomass C, and soil microbial biomass N at 0–30 cm depth. The efficiency of nutrients can be increased through the integrated use of organic manures and chemical fertilizers [32]. The slow and gradual release of N from organic manure is an advantage over sole chemical fertilization for achieving higher NUE, grain yield, and quality of rice [33,34].

Previous research in the acidic and charland soils primarily focused on the fertility assessment and the impact of combined application of manures (primarily cowdung and poultry manure) and chemical fertilizers, but not as an IPNS approach or in conjugation with conservation agriculture in other soil types [35,36,37]. The present study introduced biochar based IPNS as a means of soil nutrient management for diversified crops in these study areas for the first time. However, the present research knowledge on the impact of different IPNS approaches, especially biochar-based IPNS approaches, on crop yields, total system productivity, N uptake and use efficiency, and soil physicochemical properties under different cropping patterns is mostly limited to acidic and charland soils. We hypothesize that applying organic amendments, especially biochar, and chemical fertilizer as a means of IPNS will improve crop productivity, N uptake and use efficiency, and soil physicochemical properties. Thus, the research was conducted to (1) evaluate the effects of different IPNS approaches on soil aggregate properties; (2) determine the impacts of different IPNS approaches on soil physicochemical properties; and (3) measure the effects of different IPNS approaches on crop yields, system productivity, N uptake, and NUE.

## 2. Materials and Methods

### 2.1. Description of the Experimental Site and Initial Soil Properties

In two farmers’ fields of Madhupur, Tangail (24°59.82′ N, 90°03.99′ E) and Islampur, Jamalpur (25°80.73′ N, 89°81.90′ E), the study concentrated on acid soils in Madhupur, Tangail (24°59.82′ N, 90°03.99′ E) and charland soils in Islampur, Jamalpur (25°80.73′ N, 89°81.90′ E). The climate in the region is subtropical monsoon, with an average annual temperature of 26 °C, rainfall of 1,800 mm and an average relative humidity of 85% (local weather station). The general soil type of acid soil in Madhupur, Tangail with a deep red to brown terrace soil [38] is located in agro-ecological zone 28 (AEZ-28; Madhupur Tract), while charland soil in Islampur, Jamalpur with a noncalcareous dark grey floodplain soil [38] is located in agro-ecological zone 9 (AEZ-9; Old Brahmaputra Floodplain). The soil texture of the Madhupur site was clay loam with a pH of 5.5, making it strongly acidic, whereas the texture of the Islampur site was sandy loam with a pH of 6.6, making it near neutral in nature. Table 1 shows the initial physicochemical properties of soil at the Madhupur and Islampur sites.

### 2.2. Experimental Design and Crop Managements

At both locations, the experiment began in mid-October of 2018 and lasted through November of 2020. The experiment was set up in a randomized complete block design (RCBD, Table S1 in Supplementary Material), with the experimental area divided into four blocks representing the replications, to reduce the heterogenic effects of soil. There was a total of 24 plots in each experimental site (i.e., six treatments with four replications). The unit plot size was 5 × 4 m at both locations, with 0.75 m of interplot space, and 1 m of interblock space.

On a farmer’s field at Madhupur site (acidic soil), the treatments included PM, RHB, PMB, and dolomite (at a rate of 1 t ha^−1^ at the beginning of the experiment), whereas PM, PMB, OF, and VC were used as treatments for a farmer’s field on the charland soils of Islampur site. At both locations, organic materials were applied at a rate of 3 t ha^−1^ for each crop, biochar at a rate of 2 t ha^−1^ for each crop, while the rest of the nutrients were applied as chemical fertilizers using the IPNS approach based on agro-ecological zone (AEZ) and following the fertilizer recommendation guide-2018 (FRG-2018). The other two treatments at both sites were recommended fertilizer doses only from chemical fertilizer (RD, following FRG-2018), and no organic or inorganic fertilizer (control). The total supply of all the nutrients in each treatment except control was equivalent to the standard dose, RD. The manures had a moisture level of 15% when they were applied, and the chemical properties of the organic amendments are presented in Table 2.

At the Madhupur site (acidic soil), the cropping pattern was Mustard (*Brassica napus*)–Boro rice (Winter rice-*Oryza sativa* L.)–Transplanted (T.) Aman rice (monsoon rice-*Oryza sativa* L.). The crop varieties were BARI Sarisa-14 for mustard, BRRI dhan28 for boro rice, and BRRI dhan71 for T. Aman rice, respectively. The seed rate of mustard was 6–7 kg ha^−1^, and seeds were sown by line sowing with a distance of 25 cm between lines. Three rice seedlings were transplanted per hill with 20 × 20 cm spacing at both sites. Mustard seed was planted between mid-October and mid-November in 2018 and 2019, whereas seeds of boro rice were sown in the seedbed between 15 November and 29 November in 2018 and 2019 and transplanted in the main field from the last week of January to the first week of February in 2019 and 2020. Similarly, seeds of T. Aman rice were sown in the seedbed between 5 July and 15 July, 2019 and 2020, and transplanted by mid-August in 2019 and 2020. The recommended fertilizer doses at the Madhupur site were 90 kg N, 18 kg P, 40 kg K, 5 kg S, and 1 kg B per ha for mustard; 144 kg N, 9 kg P, 60 kg K, 4 kg S, and 1.5 kg Zn per ha for boro rice; 90 kg N, 7 kg P, 50 kg K, 4 kg S, and 1 kg Zn per ha for T. Aman rice, respectively.

Similarly, a Maize (*Zea mays*)-Jute (*Corchorus capsularis*)-T. Aman rice (monsoon rice-*Oryza sativa* L.) cropping pattern was used to carry out the experiment at the Islampur site (charland soil). The crop varieties were Kaveri 50 for maize, Tosha Paat for jute and BRRI dhan71 for T. Aman rice, respectively. Maize seed was sown in line at a rate of 25 kg ha^−1^, with a spacing of 60 × 20 cm. Similarly, the jute seed rate was 6 kg ha^−1^, and seeds were sown by line sowing with 25 × 5 cm spacing. Maize seed was sown between October and November 2018 and 2019, whereas jute seed was sown in April 2019 and 2020. T. Aman seeds were sown and transplanted at the same time as the Madhupur site. The recommended fertilizer doses at the Islampur site were 225 kg N, 40 kg P, 80 kg K, 30 kg S, 5 kg Mg, 2 kg Zn per ha for maize; 90 kg N, 8 kg P, 50 kg K, 8 kg S per ha for jute; 90 kg N, 8 kg P, 50 kg K, 4 kg S per ha for T. Aman rice, respectively.

The sources of N, P, K, S, Zn, and B were urea, triple superphosphate (TSP), muriate of potash (MoP), gypsum, zinc sulfate, and boric acid, respectively. All organic amendments, dolomite, and all chemical fertilizers except urea were applied during the land preparation for all crops. In the case of rice, urea was administered in three equal parts: 7–10 days after transplanting (DAT), 25–30 days after transplanting (DAT), and 50–55 days after transplanting (DAT). In the case of maize, one-third of the urea was applied during the land preparation and the remaining urea was separated into two equal halves. The first instalment was administered at the 8 to 10 leaf stage (30–35 days after sowing, or DAS), followed by the second instalment at 60–65 DAS. In contrast, half of the urea was provided during the final land preparation in jute and mustard. The remaining urea was applied 40–45 days after seeding in jute and during the flowering stage of mustard.

In mustard, the first irrigation was provided 20–25 days after sowing (before flowering) and the second one within 55 days of seed sowing (during fruit setting). The rice fields were irrigated a day before the final land preparation and then, when necessary, to maintain standing water at about 3 cm above the soil surface throughout the growing season. Depending on soil type and crop requirement, 4–5 irrigations were applied to maize and T. Aman, whereas boro rice required 8 irrigations during the cropping season. When the plants were 10 to 12 cm tall and produced 3–4 leaves, the jute was watered. Then, supplemental irrigation was applied to the crop in accordance with its needs.

A nonselective herbicide, glyphosate (Roundup^®^; Bayer crop science Ltd., Dhaka, Bangladesh), was sprayed across the area at a rate of 1.85 kg ha^−1^ 3 days before land preparation. Furthermore, 7 days after transplanting rice seedlings, pretilachlor (Superhit^®^, post-emergence herbicide) was applied at a rate of 450 g ha^−1^. Brifer 5G and Cidial 5G (ACI Bangladesh Ltd., Dhaka, Bangladesh) were used to control rice insects as needed. To control Alternaria leaf blight of mustard, seeds were treated with Provax-200 before sowing, and Ripcord 10 EC was sprayed to control cutworm. Jute was sprayed with Diazinon 60 EC, Karate 2.5 EC, and Dithane M-45 to prevent major diseases and pests. Contact insecticides like Ripcord 10 EC and DCC 100 EC were sprayed to control maize insects.

### 2.3. Harvesting and Data Recording

Each crop was harvested at physiological maturity in a 2 m^2^ microplot at the center of each replicated plot. For rice and mustard, grain yield was estimated at 14% moisture content and for maize it was 20%. The NUE was estimated for rice and maize only. Jute was harvested after 120 days of sowing when the flowers had been shed. During harvesting, plants were cut at ground level, and then retting, stripping, washing, sun-dried, and yield data were recorded.

### 2.4. Plant Analysis and Determination of Nitrogen Use Efficiency (NUE)

From each plot, all parts of cob (i.e., grain, outer sheath, and kernel) and stover of maize, as well as the grain and straw of rice, were chopped separately and dried in an oven at 65 °C until a constant weight was obtained. The oven-dried samples were ground and sieved in a 2-mm sieve [39]. About 0.1 g of oven dry ground plant sample was taken into a digestion flask to which 1.1 g catalyst mixture (K_2_SO_4_:CuSO_4_·5H_2_O:Se = 100:10:1) and 5 mL H_2_SO_4_ were added. The flasks were swirled and allowed to stand for about 10 min. Then heating (380 °C) was continued until the digest was clear and colorless. After cooling, the content was taken into 100 mL volumetric flask and the volume was made up to the mark with distilled water. A reagent blank was prepared in a similar manner. This digest was used for total N determination by semi micro-Kjeldahl method as described by Bremner and Mulvaney [40].

Total N uptake by crops was determined by the formula [41]:

(1) $$N\;uptake\;\left(\mathrm{kg}/\mathrm{ha}\right)=\frac{N\;content\;\left(\%\right)\;-\;Total\;dry\;mass\;production\;\left(\mathrm{kg}/\mathrm{ha}\right)}{100\;}$$

The NUE expressed as a percentage (%) was determined as described by Moll et al. [42] as follow:

(2) $$NUE\;\left(\%\right)=\frac{N\;uptake\;from\;the\;fertilized\;plot-N\;uptake\;from\;control}{Applied\;N}\times 100$$

### 2.5. Soil Sampling and Analysis

After two years of testing with the cropping patterns, Mustard-Boro-T. Aman and Maize-Jute-T. Aman, geo-referenced (GPS—recorded X, Y coordinates, and altitude) composite soils were developed under six different treatments. Firstly, composite soil samples (a mixture of five core samples) at 0–15 cm depth were collected randomly from each replicated plot after harvesting in November 2020 (6th crop phase of each sequence). Then, the samples were air-dried in the shadow for 15 days at room temperature (~25 °C).

#### 2.5.1. Soil Aggregate Properties

Soil aggregate size fractionations were performed using the wet sieving method to obtain water-stable aggregates [43] in 250 g soil over a sequence of sieves with mesh sizes 2.0, 0.85, 0.30, 0.15 and 0.053-mm. Soil aggregate fractions retained on each sieve after being dispersed in water on a planetary shaker at a rate of 31 rpm were transferred to a nickel cup and oven-dried at 65 °C until a constant weight was obtained. The respective mass of each aggregate size of soil was converted to the relative percentage (over the total mass of aggregates). Aggregate mean weight diameter (MWD) was estimated using Equation (3) below [44,45].

(3) $$MWD=\frac{\sum_{i=1}^{i=n}mi\;\times di}{\sum_{i=1}^{i=n}mi}$$

where, *mi* and *di* are weight and the mean diameter of aggregate fraction i, respectively.

Aggregate stability index (SI) was obtained by determining the MWD of air-dried soils and soils sieved after being dispersed in water on a planetary shaker [46] following Equation (4).

(4) $$Stability\;Index\;\left(SI\right)=\frac{1}{instability\;index}$$

where, Instability Index = MWD (dry sieving) − MWD (wet sieving).

#### 2.5.2. Total Nitrogen, pH and Bulk Density

Soil TN was measured using the semimicro Kjeldahl method [40]. A glass electrode pH meter was used to measure the pH of the samples in a 1:2.5 (soil:water ratio) [47]. Soil bulk density was determined by following the standard core method [48]. Antecedent soil moisture content was measured using the thermo-gravimetric method [49].

### 2.6. Statistical Analysis

A one-way analysis of variance (ANOVA) was performed using different treatments as a random variable. The distribution of data for normality was checked before ANOVA. Data were statistically analyzed to ascertain the significant differences in treatment effects using Statistix 10 software. A posthoc test was performed to separate differences between treatments using Tukey-Kamer’s multiple comparisons. All statistical analyses were considered significant at *p* < 0.05 unless otherwise mentioned.

## 3. Results

### 3.1. Effect of Different Treatments on Soil Physicochemical Properties, Crop Productivity, N Uptake and NUE in Acidic Soil

#### 3.1.1. Effect of Different Treatments on Soil Physicochemical Properties

##### Aggregate Size Distribution

**Figure 1:**
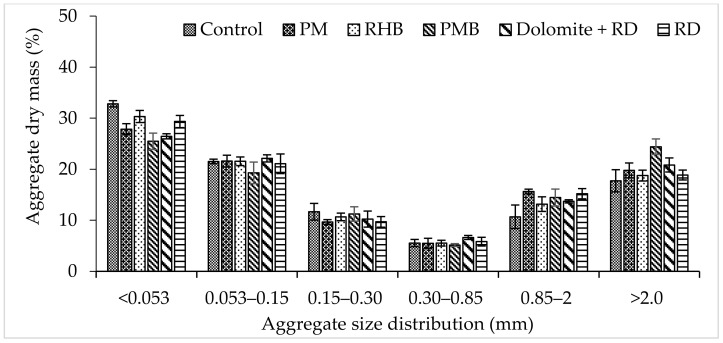
Distribution of different aggregate size fractions within 0–15 cm soil layer under different treatments in acidic soil (bars = average and line = standard error of mean, SEM; *n* = 10). (PM = poultry manure, RHB = rice husk biochar, PMB = poultry manure biochar, RD = recommended dose from only chemical fertilizer).

The proportion of soil material in aggregate size of < 0.053 mm was higher in control, which was statistically similar to RD, whereas the lowest value was observed in PMB, which was identical to all the treatments except control. The aggregate size classes of 0.053–0.15, 0.15–0.30, 0.30–0.85, and 0.85–2 mm were statistically similar in all the treatments. The aggregate size of > 2.0 mm increased due to the application of treatments compared to the control, exhibiting the highest value in PMB, although it was similar to other treatments except for control (Figure 1).

The proportion of microaggregates (<0.30 mm) was greater in all the treatments than macroaggregates (>0.30) (Table 3). The highest microaggregate proportion (%) was observed in control, which was statistically similar to RD, whereas treatments except control were similar. In contrast, macroaggregate increased in all the treatments compared to the control. MWD, GMD, and SI increased due to the application of different treatments compared to the control treatment. All the treatments except control were similar to each other with respect these properties (Table 3).

##### Total Nitrogen, pH, and Bulk Density

Different treatments had a significant influence on soil TN (*p* < 0.001, Table 2). Except for RHB, all the treatments significantly increased TN content in the soil compared to that of the control. The highest TN increase over control was observed in PMB (33.3%), whereas the least increase was observed in RD (11.1%) (Table 4). Likewise, soil pH was also significantly influenced by different treatments (*p* < 0.001, Table 4). Soil pH ranged from 5.22 to 5.72 in various treatments. The highest soil pH was observed in PMB, which was identical to PM, whereas the lowest value was observed in Dolomite + RD and RD. Soil pH decreased by 3.3 and 3.5% in RD and Dolomite + RD, respectively, compared to that of the control, whereas soil pH increased by 2.4, 3.0, and 5.7%, respectively, in RHB, PM, and PMB, respectively. In contrast, different treatments had no significant effect on soil BD (*p* > 0.05, Table 4).

#### 3.1.2. Effect of Different Treatments on Crop Yield and System Productivity of Mustard-Boro-T. Aman Rice Cropping Pattern

Different IPNS treatments significantly influenced grain yield of mustard in both 2019 and 2020, and straw yield in 2020 (*p* < 0.001, Table 5). In 2019, the increase in grain yield of mustard over control varied from 66.7 to 116.7%. The highest increase was observed in PMB and RD, and the lowest increase in PM and Dolomite + RD. In 2020, the increases in grain and straw yields of mustard over control varied from 85.7 to 128.67% and 79.2 to 199.9%, respectively. The highest increases were observed in Dolomite + RD, and the lowest increases in PMB (Table 5).

Likewise, grain and straw yield of boro rice were also significantly influenced by different IPNS treatments in both 2019 and 2020 (*p* < 0.001, Table 5). In 2019, the increases in grain and straw yields of boro rice over control ranged from 47.5 to 67.5% and 41.7 to 59.3%, respectively. The highest increases were observed in RHB, and the lowest increases in Dolomite + RD. In 2020, the increases in grain and straw yields of boro rice over control varied from 80.0 to 102.9% and 64.5 to 77.3%, respectively. The highest increases in grain and straw yields were observed in PMB and RHB, respectively, and the lowest increases in RHB and RD for grain and straw yields, respectively (Table 5).

Similarly, grain and straw yield of T. Aman rice were also significantly influenced by different IPNS treatments in both 2019 and 2020 (*p* < 0.001, Table 5). In 2019, the increases in grain and straw yields of T. Aman rice over control varied from 50.0 to 66.7% and 38.7 to 50.3%, respectively. The highest increases in grain and straw yields were observed in Dolomite + RD, and the lowest increase in PM. In 2020, the increases in grain and straw yields of T. Aman rice over control varied from 42.9 to 60.0% and 16.1 to 34.3%, respectively. The highest increases were observed in RHB, and the lowest increases in RD (Table 5).

Different IPNS treatments significantly increased the total system productivity of the Mustard-Boro-T. Aman rice cropping pattern compared to that of the control in 2019 and 2020 (*p* < 0.001, Table 5). In 2019, the increase in system productivity in different treatments over control ranged from 55.4 to 69.6%, exhibiting the highest value in PMB and the lowest in Dolomite + RD (Table 5). In 2020, the increase over control varied from 74.7 to 82.8%. The highest increase was observed in Dolomite + RD and the lowest in RHB (Table 5).

#### 3.1.3. Effect of Different Treatments on Nitrogen Uptake and NUE by T. Aman and Boro Rice

N uptake and NUE by T. Aman rice were also significantly influenced by different IPNS treatments in both 2019 and 2020 (Table 6). In 2019, the increase in N uptake over control from 45.1 to 63.4% exhibited the highest PMB value and the lowest RHB value (Table 3). The increase in N uptake over control ranged from 54.1 to 65.9% in 2020. The highest increase was observed in PMB and the lowest increase was observed in PM and RD, respectively (Table 6). The highest NUE was noted in PMB in both 2019 and 2020, whereas the lowest NUE was noted in RHB and RD, respectively, in 2019 and 2020 (Table 6).

Similarly, different IPNS treatments significantly influenced N uptake by boro rice in 2019 and 2020, and NUE in 2020 (Table 6). In 2019, the increase in N uptake over that of control ranged from 65.7 to 74.7%, exhibiting the highest RHB value and the lowest RD value (Table 3). The increase in N uptake over control ranged from 76.4 to 96.2% in 2020. The highest increase was observed in PMB and the lowest increase was observed in RHB (Table 6). The highest NUE was noted in PMB in 2019 and 2020, whereas the lowest was noted in Dolomite + RD and RHB in 2019 and 2020 (Table 6).

### 3.2. Effect of Different Treatments on Soil Physicochemical Properties, Crop Productivity, N Uptake and NUE in Charland Soil

#### 3.2.1. Effect of Different Treatments on Soil Physicochemical Properties

##### Aggregate Size Distribution

**Figure 2:**
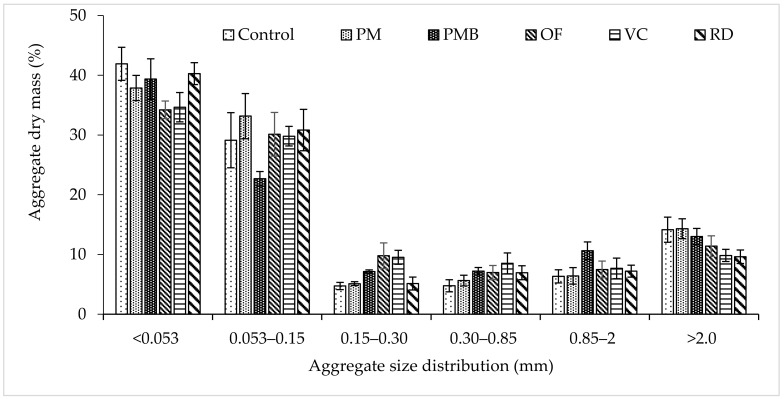
Distribution of different aggregate size fractions within 0–15 cm soil layer under different treatments in charland soil (bars = average and line = standard error of mean, SEM; *n* = 10). (PM = poultry manure, PMB = poultry manure biochar, OF = compost, VC = vermicompost, RD = recommended dose from only chemical fertilizer).

The proportion of soil material in aggregate size classes of < 0.053 was higher in control, which was statistically similar to RD, PM, and VC, whereas the lowest value was observed in PMB, which was identical to OF and VC. The aggregate size classes of 0.053-0.15, 0.15–0.30, and 0.30–0.85 mm were statistically similar in all the treatments (Figure 2). The highest aggregate size of 0.85–2.0 mm was observed in PMB, which was statistically identical to OF (Figure 2). Similarly, the proportion of aggregate size of > 2.0 mm also increased when the soil was treated with PMB based IPNS, which was statistically similar to PM (Figure 2).

The proportion of micro aggregates (< 0.30 mm) were greater in all the treatments than macroaggregates (> 0.30) (Table 7). The highest micro aggregate proportion (%) was observed in RD which was statistically similar to all the treatments except PMB. In contrast, macro aggregate increased in PMB compared to that of the control, other treatments were statistically identical. MWD, GMD, and SI increased by 32, 11, and 19% in PMB compared to that of the control treatment. All the treatments except PMB were similar to control in all these cases (Table 7).

##### Total Nitrogen, pH, and Bulk Density

Organic amendment based IPNS approaches had no significant effects on soil TN and pH after the completion of the experiment (*p* > 0.05, Table 8). In contrast, different treatments in this study significantly influenced soil BD (*p* < 0.05, Table 8). Soil BD ranged from 1.26 to 1.33 g cm^−3^, exhibiting the highest value in control and RD, identical to other treatments except for PMB. The lowest value was observed in PMB, which was identical to PM and OF, respectively. Compared to that of control, BD decreased by about 0.8, 2.3, 3.0, and 5.3% in VC, PM, OF, and PMB, respectively.

#### 3.2.2. Effect of Different Treatments on Crop Yield and System Productivity of Maize-Jute-T. Aman Rice Cropping Pattern

Different IPNS treatments significantly influenced grain and straw yields of maize in both 2019 and 2020 (*p* < 0.001, Table 9). In 2019, the increases in grain and straw yields of maize over control varied from 60.9 to 89.1% and 99.1 to 138.9%, respectively. The highest increases in grain and straw yields were observed in PM, and the lowest increases in grain and straw yields were observed in VC and PMB, respectively. In 2020, the increases in grain and straw yields of maize over control varied from 106.1 to 130.3% and 53.5 to 83.1%, respectively. The highest increases in grain and straw yields were observed in PMB and PM, respectively, and the lowest increases in VC (Table 9).

Likewise, the fiber yield of jute was also significantly influenced by different IPNS treatments in both 2019 and 2020 (*p* < 0.001, Table 9). In 2019, jute yield in different treatments ranged from 2.5 to 3.7 t ha^−1^, exhibiting the highest yield in VC, which was identical to all the treatments except control, and the lowest yield in control. The increase in yield over control varied from 24.0 to 48.0%. The highest increase was observed in VC, and the lowest increase in RD. In 2020, jute yield in different treatments ranged from 1.2 to 2.6 t ha^−1^, exhibiting the highest yield in PMB, which was similar to PM, and the lowest yield in control. The increase in yield over control varied from 33.3 to 116.7%. The highest increase was observed in PMB, and the lowest increase in OF and RD (Table 9).

Similarly, grain and straw yields of T. Aman rice were also significantly influenced by different IPNS treatments in both 2019 and 2020 (*p* < 0.001, Table 9). In 2019, the increases in grain and straw yield of T. Aman over control ranged from 24.3 to 45.9% and 22.1 to 31.7%, respectively. The highest increases in grain and straw yields were observed in OF, and the lowest increases in PM. In 2020, the increase in grain and straw yields of T. Aman rice over control varied from 77.8 to 138.9% and 94.9 to 124.3%, respectively. The highest increases in grain and straw yields were observed in OF and PMB, respectively, and the lowest increases in grain and straw yields were observed in VC and PM, respectively (Table 9).

Different IPNS treatments significantly increased the total system productivity of the Maize-Jute-T. Aman rice cropping pattern compared to that of the control in 2019 and 2020 (*p* < 0.001, Table 9). In 2019, the increase in system productivity in different treatments over control ranged from 43.3 to 51.1%, exhibiting the highest value in PMB and the lowest in RD (Table 9). In 2020, the increase over control varied from 74.4 to 115.4%. The highest increase was observed in PMB and the lowest in VC.

#### 3.2.3. Effect of Different Treatments on N Uptake and NUE by T. Aman Rice and Maize

N uptake and NUE by T. Aman rice were also significantly influenced by different IPNS treatments in both 2019 and 2020 (Table 10). In 2019, the increase in N uptake over control from 35.5 to 47.2% exhibited the highest value in OF and the lowest value in PM (Table 10). On the other hand, the increase in N uptake over control ranged from 87.1 to 109.3% in 2020. The highest increase was observed in OF and the lowest increase was observed in PM (Table 10). The highest NUE was noted in PMB and the lowest NUE was noted in PM in both 2019 and 2020, respectively (Table 10).

Similarly, different IPNS treatments significantly influenced N uptake and NUE by maize in both 2019 and 2020 (Table 10). In 2019, the increase in N uptake over control ranged from 71.5 to 79.2%, exhibiting the highest value in OF and the lowest value in VC (Table 10). The increase in N uptake over control ranged from 94.5 to 105.7% in 2020. The highest increase was observed in OF and the lowest increase was observed in PM (Table 10). The highest NUE was noted in OF in both 2019 and 2020, whereas the lowest NUE was noted in VC and PM, in both 2019 and 2020 (Table 10).

## 4. Discussion

### 4.1. Effect of IPNS on Soil Physicochemical Properties

IPNS is used worldwide to improve the physical, chemical, and biological properties of degraded soils. The IPNS treatments in the present study significantly influenced the soil physicochemical properties of the acidic and charland soils. Our results demonstrated that all the IPNS treatments, particularly biochar and compost-based IPNS, significantly increased water-soluble macroaggregate (WSA) groups in soils, stability index, MWD, and GMD (Table 3 and Table 7), which were in accordance with previous studies [50,51,52]. Biochar and compost, according to Ibrahim et al. [53], significantly improved aggregate stability and water-soluble macroaggregate (>0.25 mm) in loamy sand soil. Mi et al. [54] found that NPK and organic matter (straw residue or cattle manure) significantly increased the water-soluble aggregate > 0.25 mm, MWD, and GMD, while reducing the proportion of 0.25 mm aggregates at two field experiment sites in Jinhua, Zhejiang province, and Jintan, Jiangsu province. According to Annabi et al. [55], adding organic matter to loam soils affects aggregate stability, and they ascribe this to increased microbial activity, which improves aggregate stability. In research conducted by Šimanský et al. [56], using N fertilizer in combination with biochar increased the macroaggregates. They observed that WSA of 3–2 mm and 5–3 mm sizes were 75 and 149% higher when biochar was applied along with N fertilizer (20 t biochar ha^−1^ + 80 kg N ha^−1^), whereas sole application of biochar (20 t ha^−1^) resulted in a comparatively small (0.5–0.25 mm) size aggregate. Similar results were also observed by Ma et al. [57], who reported that both the NPK fertilizer + maize straw and NPK + biochar treatment significantly enhanced the relative proportion of macroaggregates (> 2 mm) and the mean weight diameter while decreasing the relative ratio of microaggregates (< 0.25 mm). Organic matter is essential for repositioning soil particles so that aggregates can form [58]. Organic material incorporation into soil considerably increases soil organic matter content while also releasing organic acids, polysaccharides, and other by products that bind free primary particles into macroaggregates, resulting in the formation of macroaggregates with a larger (> 2 mm) size [59]. Another assumption is that organic manures function as stimulants for microbes [60], encouraging microbial development and increasing the density and efficacy of mycorrhizal hyphae, which enhances aggregate stability [61,62,63]. Adding nitrogen to the soil can speed up the biochar mineralization process, resulting in increased aggregation [56,64]. However, Xin et al. [65] reported that integrated application of half organic compost and half mineral fertilizer did not result in greater proportions of the > 0.25 mm aggregate than after using NPK for 23 years. Biochar made from a mixture of wood and straw, on the other hand, did not influence aggregate stability [55]. Furthermore, due to differences in parameters such as the quality, amount, and timing of organic matter addition, the research does not clearly show a link between aggregate stability and rates of organic input [66].

Addition of organic and inorganic fertilizers significantly increased soil TN in acidic soil (Table 4), which aligns with Islam et al. [7,24,25] and Van Chuong [67]. Previous research showed that the C:N ratio of the manure is essential for mineralization and the build-up of SOC and TN, and that a low C:N ratio boosts the mineralization process, resulting in increased soil TN [68,69]. However, the effect of IPNS on soil TN in charland soil was nonsignificant (Table 8). Our results suggest that IPNS is more efficient in acidic soil than in charland soil in terms of increasing soil TN.

Our results demonstrated that IPNS treatments significantly increased soil pH compared to the control, although sole application of chemical fertilizer decreased soil pH, as supported by previous studies [54,70,71]. Lin et al. [72] observed that organic fertilizer and chemical fertilizer significantly increased the tea orchard soil pH and microbial population. This is attributed to the increased soil organic matter content, which encourages soil maturation, improves soil structure, and raises the soil base saturation percentage by the organic manure, which can help to reduce soil acidity [73,74]. On the other hand, chemical fertilizer undergoes rapid hydrolysis and subsequent nitrification, resulting in the release of protons and a decrease in soil pH.

The results revealed that IPNS treatments had no significant effect on soil bulk density in acidic soil, although IPNS treatments significantly decreased soil bulk density in charland soil. Many studies have shown that including organic material in the soil reduces its bulk density [75,76]. The reason for the decreased soil bulk density is the addition of organic materials, which increases soil organic matter content, resulting in soils that are more friable, porous, and chemically active, reducing soil bulk density [77]. However, in acidic soil, the effect was not observed, which may be due to the clay type soil texture.

### 4.2. Effect of IPNS on Crop Yield, Nitrogen Uptake and Nitrogen Use Efficiency

Chemical fertilizers are widely used in the current agricultural farming system, which has a detrimental impact on soil health, the environment, and crop yield. Nitrogen is the most important yet limited nutrient among chemical fertilizers worldwide due to very low NUE due to gaseous losses such as denitrification, volatilization, and leaching from agricultural cropping systems [78,79]. Our results showed that the combined application of organic manure, inorganic fertilizer and dolomite significantly increased total crop yields (i.e., system productivity) of the cropping patterns (Table 5 and Table 9), which is consistent with previous studies in acidic soil [7,24,25]. In the acidic soil of Nalitabari, Sherpur, Islam et al. [7,24] reported that combined application of lime, organic amendment, and chemical fertilizer increased yield and nutrient uptake of the T. Aman-Maize-Fallow and Wheat-Mungbean-T. Aman cropping patterns. In another study at the same site as the present study, Islam et al. [25] observed that combined application of lime, organic manure, and chemical fertilizer significantly improved the total system productivity and nutrient absorption of the T. Aman-Mustard-Boro cropping pattern with subsequent improvement in the soil quality. The authors postulated the rise in soil pH and other soil physicochemical properties as the cause of the increase in crop productivity and nutrient uptake. Iqbal et al. [80] reported that 30% PM + 70% chemical fertilizer increased rice grain yield by 95% and nutrient uptake and NUE. In a field experiment at the Agronomic Research Farm, University of Agriculture Faisalabad, Pakistan, Mahmood et al. [81] observed that NPK at 150-85-50 + 7 t ha^−1^ PM significantly increased maize yield with concurrent improvement in soil properties. Similarly, Bedada et al. [82] found that the combined application of compost and urea improved soil physicochemical properties and increased maize production. Improvement in soil physical conditions along with a sufficient amount of water enhanced crop nutrient uptake capacity, resulting in greater biological output at a specific quantity of fertilizer application [83]. Hossain et al. [84] similarly found that FYM treatment increased N absorption in rice compared to no fertilizer and inorganic fertilizer applications. Chemical fertilizer alone reduced nutrient absorption due to poor availability and more significant nutritional loss [85]. Crop N requirements must be synchronized with N supply to improve N usage efficiency. Crop type, availability of other nutrients, nutrient leaching, weather, and genotypic variations are all factors that influence crop NUE [86,87]. In rice, Dawe et al. [88] discovered that N losses are reduced as yield and NUE increase, while Fofana et al. [89] observed comparable effects in maize crops. In two-year field research, Rehim et al. [90] reported a 98% increase in N absorption of wheat grains and a 200% increase in NUE when 75% urea+25% farmyard manure was applied instead of urea alone. These findings showed that combining organic and inorganic sources to improve NUE in crops is more effective [91].

## 5. Conclusions

Two-year evaluation of IPNS indicates that it is potentially a good practice for sustainable crop production in acidic and charland soils. The present research results revealed that poultry manure biochar-based IPNS showed the best enrichments in terms of crop yields, system productivity, nitrogen uptake, and NUE of the Mustard-Boro-T. Aman cropping pattern in acidic soil. All the IPNS treatments improved soil aggregate properties, with increased macroaggregate formation, and also enhanced soil TN concentration and pH. Compost and poultry manure biochar based IPNS improved the crop yields, total system productivity, nitrogen uptake, and use efficiency of the Maize-Jute-T. Aman cropping pattern in charland soil. Poultry manure biochar based IPNS increased macroaggregate and decreased the soil bulk density of charland soil. Therefore, poultry manure biochar- and compost-based IPNS can be recommended for maximizing crop productivity, nitrogen uptake, and use efficiency, with subsequent improvement of the physicochemical properties of acidic and charland soils.

## Figures and Tables

**Table 1:** Initial soil properties for acid soil and charland soil.

Characteristics	Acidic Soil	Charland Soil
Texture	Clay Loam	Sandy Loam
SOC (g kg^−1^)	9.9	5.3
TN (g kg^−1^)	0.9	0.5
Available P (mg kg^−1^)	17.63	12.37
Available S (mg kg^−1^)	14.95	10.53
Available K (cmolc kg^−1^)	0.12	0.12
pH	5.5	6.6
CEC (cmolc kg^−1^)	17.71	10.31
Moisture (%)	21.69	17.28
Bulk Density (g cm^−3^)	1.15	1.32

**Table 2:** Chemical properties of organic amendments used in experiments.

Organic Amendments	OC (g kg^−1^)	TN (g kg^−1^)	pH	CEC (cmolc kg^−1^)	Available P (mg kg^−1^)	Available K (cmolc kg^−1^)	Available S (mg kg^−1^)
PM	85.6	20.5	8.3	12.29	839	6.34	1898
VC	75.7	10.8	7.7	11.83	1020	4.99	377
OF	72.7	11.2	7.3	10.07	983	5.47	1469
RHB	175.2	18.1	7.5	19.54	1149	15.99	415
PMB	337.6	30.8	8.5	35.68	1437	22.61	2094

PM = Poultry Manure, VC = Vermicompost, OF = compost, RHB = Rice Husk Biochar, PMB = Poultry Manure Biochar.

**Table 3:** Effects of different treatments on soil aggregate properties of acidic soil.

Treatment	MaAS (%)	MiAS (%)	SI (ratio)	MWD (mm)	GMD (mm)
Control	33.97 ± 1.50 b	66.03 ± 1.50 a	0.52 ± 0.03 b	0.60 ± 0.03 b	0.22 ± 0.01 b
PM	40.92 ± 0.76 a	59.08 ± 0.76 b	0.69 ± 0.02 ab	0.71 ± 0.02 ab	0.27 ± 0.01 ab
RHB	40.94 ± 0.75 a	59.06 ± 0.75 b	0.69 ± 0.02 ab	0.72 ± 0.01 ab	0.28 ± 0.01 ab
PMB	44.00 ± 2.21 a	56.00 ± 2.21 b	0.79 ± 0.07 a	0.78 ± 0.04 a	0.32 ± 0.03 a
Dolomite + RD	41.19 ± 1.70 a	58.81 ± 1.70 b	0.70 ± 0.05 ab	0.71 ± 0.03 ab	0.28 ± 0.01 ab
RD	39.89 ± 0.47 ab	60.11 ± 0.47 ab	0.66 ± 0.01 ab	0.69 ± 0.01 ab	0.26 ± 0.00 ab
Level of significance	**	**	*	**	**

PM = poultry manure, RHB = rice husk biochar, PMB = poultry manure biochar, RD = recommended dose from only chemical fertilizer, MaAS = proportional macroaggregate mass, MiAS = proportional microaggregate mass, SI = stability index, MWD = aggregate mean weight diameter, GMD = aggregate geometric mean diameter. Data are mean ± SE (*n* = 4). Averaged values within a column, succeeded by different small letters (a, b) differ significantly, * indicates significant at 5% level of significance, ** indicates significant at 1% level of significance.

**Table 4:** Effects of different treatments on physicochemical properties of acidic soil.

Treatment	TN (%)	pH (H_2_O)	BD (g cm^−3^)
Control	0.09 ± 0.00 bc	5.41 ± 0.03 b	1.25 ± 0.02
PM	0.11 ± 0.00 ab	5.57 ± 0.04 ab	1.15 ± 0.03
RHB	0.09 ± 0.00 c	5.54 ± 0.03 b	1.22 ± 0.02
PMB	0.12 ± 0.00 a	5.72 ± 0.02 a	1.18 ± 0.03
Dolomite + RD	0.11 ± 0.00 a	5.22 ± 0.04 c	1.23 ± 0.03
RD	0.10 ± 0.01 abc	5.23 ± 0.04 c	1.24 ± 0.04
Level of significance	***	***	ns

PM = poultry manure, RHB = rice husk biochar, PMB = poultry manure biochar, RD = recommended dose from only chemical fertilizer. Data are mean ± SE (*n* = 4). Averaged values within a column, succeeded by different small letters (a, b, c), differ significantly between different treatments at *p* < 0.05 significance level. *** indicates significant at the 0.001 probability level, ns = non-significant.

**Table 5:** Effects of different treatments on crop yield and system productivity of Mustard-Boro-T. Aman rice cropping pattern in acidic soil.

Grain Yield								
	Mustard		Boro		T. Aman		System Productivity	
	2019	2020	2019	2020	2019	2020	2019	2020
Control	0.6 ± 0.02 d	0.7 ± 0.02 d	4.0 ± 0.10 c	3.5 ± 0.35 c	3.6 ± 0.27 c	3.5 ± 0.45 b	9.2 ± 0.27 d	8.7 ± 0.25 b
PM	1.0 ± 0.01 c	1.4 ± 0.10 bc	6.5 ± 0.18 a	6.6 ± 0.28 ab	5.4 ± 0.11 b	5.2 ± 0.47 a	14.4 ± 0.31 bc	15.3 ± 0.83 a
RHB	1.1 ± 0.01 b	1.3 ± 0.16 c	6.7 ± 0.17 a	6.3 ± 0.42 b	5.5 ± 0.13 ab	5.6 ± 0.28 a	15.0 ± 0.19 abc	15.2 ± 0.31 a
PMB	1.3 ± 0.01 a	1.3 ± 0.10 c	6.6 ± 0.04 a	7.1 ± 0.20 a	5.7 ± 0.11 ab	5.5 ± 0.06 a	15.6 ± 0.13 a	15.8 ± 0.13 a
Dolomite + RD	1.0 ± 0.01 c	1.6 ± 0.07 a	5.9 ± 0.25 b	6.5 ± 0.38 ab	6.0 ± 0.09 a	5.4 ± 0.22 a	14.3 ± 0.10 c	15.9 ± 0.41 a
RD	1.3 ± 0.01 a	1.5 ± 0.25 ab	6.2 ± 0.39 ab	6.6 ± 0.23 ab	5.6 ± 0.10 ab	5.0 ± 0.13 a	15.1 ± 0.23 ab	15.5 ± 0.19 a
Level of significance	***	***	***	***	***	***	***	***
**Straw Yield**								
**Treament**	**Mustard**		**Boro**		**T. Aman**			
	**2019**	**2020**	**2019**	**2020**	**2019**	**2020**		
Control	1.1 ± 0.06 a	0.9 ± 0.13 b	4.4 ± 0.13 a	4.0 ± 0.40 b	4.3 ± 0.32 b	4.6 ± 0.36 b		
PM	1.9 ± 0.39 a	2.2 ± 0.21 a	6.7 ± 0.16 b	7.0 ± 0.15 a	6.0 ± 0.17 a	5.8 ± 0.50 ab		
RHB	1.5 ± 0.34 a	2.0 ± 0.41 ab	6.9 ± 0.09 b	7.1 ± 0.44 a	6.4 ± 0.12 a	5.8 ± 0.45 ab		
PMB	1.1 ± 0.09 a	1.6 ± 0.12 ab	6.9 ± 0.09 b	7.1 ± 0.08 a	6.5 ± 0.20 a	6.1 ± 0.15 a		
Dolomite + RD	1.6 ± 0.20 a	2.7 ± 0.12 a	6.2 ± 0.30 b	6.8 ± 0.19 a	6.5 ± 0.16 a	6.1 ± 0.04 a		
RD	1.9 ± 0.24 a	1.9 ± 0.40 ab	6.4 ± 0.36 b	6.6 ± 0.17 a	6.5 ± 0.30 a	5.3 ± 0.24 ab		
Level of significance	ns	**	***	***	***	*		

PM = poultry manure, RHB = rice husk biochar, PMB = poultry manure biochar, RD = recommended dose from only chemical fertilizer, Data are mean ± SE (*n* = 4). Averaged values within a column, succeeded by different small letters (a, b, c, d), differ significantly between different treatments at *p* < 0.05 significance level. * Significant at the 0.05 probability level. ** Significant at the 0.01 probability level. *** Significant at the 0.001 probability level, ns = nonsignificant.

**Table 6:** Effects of different treatments on nitrogen uptake and NUE in acidic soil.

Treatment	N Uptake (kg ha^−1^)				NUE (%)			
	Aman 2019	Aman 2020	Boro 2019	Boro 2020	Aman 2019	Aman 2020	Boro 2019	Boro 2020
Control	54.1 ± 3.5 c	53.4 ± 3.9 b	57.4 ± 2.9 b	52.6 ± 2.5 c				
PM	83.7 ± 0.6 ab	82.3 ± 4.4 a	97.7 ± 3.2 a	93.4 ± 1.5 b	32.8 ± 0.8 a	32.1 ± 4.9 ab	33.6 ± 2.7	34.0 ± 1.25 b
RHB	78.5 ± 2.08 b	83.7 ± 4.7 a	100.3 ± 3.9 a	92.8 ± 1.1 b	27.1 ± 2.3 b	33.6 ± 5.3 b	35.7 ± 3.3	33.5 ± 0.92 b
PMB	88.4 ± 1.04 a	88.6 ± 2.3 a	102.4 ± 0.3 a	103.2 ± 1.0 a	38.1 ± 1.2 a	39.0 ± 2.6 a	37.5 ± 0.3	42.2 ± 0.9 a
Dolomite + RD	82.2 ± 1.5 ab	83.6 ± 3.02 a	95.1 ± 2.1 a	95.2 ± 2.1 ab	31.2 ± 1.7 b	33.5 ± 3.4 ab	31.4 ± 1.8	35.5 ± 1.8 b
RD	82.2 ± 0.5 ab	82.3 ± 1.8 a	96.8 ± 1.07 a	94.3 ± 1.3 b	31.2 ± 0.6 b	32.0 ± 2.0 ab	32.8 ± 0.9	34.8 ± 1.1 b
Level of signific ance	***	***	***	***	**	*	ns	**

PM = poultry manure, RHB = rice husk biochar, PMB = poultry manure biochar, RD = recommended dose from only chemical fertilizer, Data are mean ± SE (*n* = 4). Averaged values within a column, succeeded by different small letters (a, b, c) differ significantly, ns indicates nonsignificant, * indicates significant at 5% level of significance, ** indicates significant at 1% level of significance, *** indicates significant at 0.1% level of significance.

**Table 7:** Effects of different treatments on soil aggregate properties of charland soil.

Treatment	MaAS (%)	MiAS (%)	SI (Ratio)	MWD (mm)	GMD (mm)
Control	25.24 ± 2.88 b	74.76 ± 2.88 a	0.34 ± 0.05 b	0.46 ± 0.05 b	0.16 ± 0.01 b
PM	26.36 ± 2.05 b	76.14 ± 1.97 a	0.35 ± 0.03 b	0.48 ± 0.04 b	0.16 ± 0.01 b
PMB	30.83 ± 2.26 a	69.17 ± 2.26 b	0.45 ± 0.05 a	0.51 ± 0.04 a	0.19 ± 0.02 a
OF	25.86 ± 1.68 b	74.14 ± 1.68 a	0.35 ± 0.03 b	0.45 ± 0.03 b	0.17 ± 0.01 b
VC	26.02 ± 3.26 b	73.98 ± 3.26 a	0.36 ± 0.06 b	0.43 ± 0.04 b	0.17 ± 0.02 b
RD	23.76 ± 1.45 b	76.24 ± 1.45 a	0.31 ± 0.02 b	0.40 ± 0.02 b	0.15 ± 0.01 b
Level of signific ance	***	***	***	***	***

PM = poultry manure, PMB = poultry manure biochar, OF = compost, VC = vermicompost, RD = recommended dose from only chemical fertilizer, MaAS = proportional macro aggregate mass, MiAS = proportional micro aggregate mass, SI = stability index, MWD = aggregate mean weight diameter, GMD = aggregate geometric mean diameter. Data are mean ± SE (*n* = 4). Averaged values within a column, succeeded by different small letters (a, b), differ significantly between different treatments at *p* < 0.05 significance level. *** Significant at 0.001 probability level.

**Table 8:** Effects of different treatments on physicochemical properties of charland soil.

Treatment	TN (%)	pH (H_2_O)	BD g cm^−3^
Control	0.07 ± 0.01	6.43 ± 0.11	1.33 ± 0.05 a
PM	0.07 ± 0.01	6.53 ± 0.13	1.30 ± 0.00 ab
PMB	0.07 ± 0.00	6.55 ± 0.05	1.26 ± 0.05 b
OF	0.07 ± 0.01	6.59 ± 0.09	1.29 ± 0.06 ab
VC	0.07 ± 0.01	6.43 ± 0.08	1.32 ± 0.04 a
RD	0.06 ± 0.00	6.47 ± 0.14	1.33 ± 0.06 a
Level of significance	ns	ns	*

PM = poultry manure, PMB = poultry manure biochar, OF = compost, VC = vermicompost, RD = recommended dose from only chemical fertilizer, Data are mean ± SE (*n* = 4). Averaged values within a column, succeeded by different small letters (a, b) differ significantly, * indicates significant at 5% level of significance, ns indicates nonsignificant.

**Table 9:** Effects of different treatments on crop yield and system productivity of Maize-Jute-T. Aman rice cropping pattern in charland soil.

Grain Yield								
Treatment	Maize		Jute		T. Aman		System Productivity	
	2019	2020	2019	2020	2019	2020	2019	2020
Control	4.6 ± 0.02 c	3.3 ± 0.31 d	2.5 ± 0.26 b	1.2 ± 0.30 c	3.7 ± 0.11 d	1.8 ± 0.18 d	14.1 ± 0.31 b	7.8 ± 0.43 d
PM	8.7 ± 0.02 a	7.1 ± 0.53 bc	3.3 ± 0.29 ab	2.3 ± 0.33 ab	4.6 ± 0.29 c	3.4 ± 0.23 c	20.5 ± 0.37 a	15.4 ± 0.61 ab
PMB	7.9 ± 0.02 ab	7.6 ± 0.25 a	3.6 ± 0.39 a	2.6 ± 0.18 a	5.4 ± 0.19 ab	3.5 ± 0.22 bc	21.3 ± 0.45 a	16.8 ± 0.44 a
OF	8.0 ± 0.01 ab	7.0 ± 0.29 bc	3.4 ± 0.38 ab	1.6 ± 0.11 bc	5.4 ± 0.12 a	4.3 ± 0.11 a	21.1 ± 0.59 a	14.6 ± 0.26 bc
VC	7.4 ± 0.03 b	6.8 ± 0.50 c	3.7 ± 0.41 a	1.7 ± 0.05 bc	4.8 ± 0.03 bc	3.2 ± 0.09 c	20.5 ± 0.72 a	13.6 ± 0.15 c
RD	8.1 ± 0.03 ab	7.2 ± 0.15 b	3.1 ± 0.15 ab	1.6 ± 0.16 bc	5.2 ± 0.24 ab	3.9 ± 0.28 ab	20.2 ± 0.43 a	14.3 ± 0.28 bc
Level of significance	***	***	*	***	***	***	***	***
**Straw Yield**								
**Treatment**	**Maize**		**T. Aman**					
	**2019**	**2020**	**2019**	**2020**				
Control	5.5 ± 0.03 e	9.0 ± 0.27 b	4.4 ± 0.13 b	2.3 ± 0.25 a				
PM	11.9 ± 0.03 b	16.2 ± 0.45 a	5.4 ± 0.25 ab	4.4 ± 0.35 b				
PMB	13.1 ± 0.17 a	15.8 ± 0.41 a	5.7 ± 0.36 a	5.1 ± 0.47 b				
OF	10.9 ± 0.02 d	15.5 ± 0.19 a	5.8 ± 0.24 a	4.9 ± 0.07 b				
VC	11.3 ± 0.05 c	14.4 ± 0.82 a	5.6 ± 0.03 a	4.4 ± 0.10 b				
RD	12.8 ± 0.05 a	16.3 ± 0.25 a	5.8 ± 0.11 a	4.5 ± 0.31 b				
Level of significance	***	***	**	***				

PM = poultry manure, PMB = poultry manure biochar, OF = compost, VC = vermicompost, RD = recommended dose from only chemical fertilizer, Data are mean ± SE (*n* = 4). Averaged values within a column, succeeded by different small letters (a, b, c, d), differ significantly between different treatments at *p* < 0.05 significance level. * Significant at 0.05 probability level. ** Significant at 0.01 probability level. *** Significant at 0.001 probability level.

**Table 10:** Effects of organic amendments on N uptake and N use efficiency by T. Aman rice and maize.

Treatment	N Uptake (kg ha^−1^)				NUE (%)			
	Aman 2019	Aman 2020	Maize 2019	Maize 2020	Aman 2019	Aman 2020	Maize 2019	Maize 2020
Control	67.1 ± 3.7 c	31.1 ± 0.8 d	97.8 ± 3.7 b	72.1 ± 1.2 c				
PM	90.9 ± 0.9 b	58.2 ± 0.8 c	169.5 ± 1.5 a	140.2 ± 0.5 b	26.4 ± 1.0 c	30.1 ± 1.0 c	32.6 ± 0.7 b	31.0 ± 0.2 c
PMB	95.4 ± 0.5 ab	60.2 ± 0.4 bc	170.0 ± 0.4 a	144.0 ± 1.3 b	31.5 ± 0.6 b	32.3 ± 0.5 bc	32.8 ± 0.2 ab	32.7 ± 0.6 b
OF	98.8 ± 0.6 a	65.1 ± 0.2 a	175.3 ± 0.6 a	148.3 ± 0.2 a	35.3 ± 0.7 a	37.7 ± 0.3 a	35.3 ± 0.3 a	34.6 ± 0.1 a
VC	96.2 ± 0.3 ab	59.8 ± 0.6 bc	167.7 ± 1.3 a	143.7 ± 0.2 b	32.4 ± 0.4 b	31.9 ± 0.8 bc	31.8 ± 0.6 b	32.6 ± 0.1 b
RD	96.0 ± 0.3 ab	61.8 ± 0.7 b	170.2 ± 1.1 a	144.3 ± 0.6 ab	32.2 ± 0.4 b	34.1 ± 0.8 b	32.9 ± 0.5 ab	32.8 ± 0.3 b
Level of significance	***	***	***	***	***	***	**	***

PM = poultry manure, PMB = poultry manure biochar, OF = compost, VC = vermicompost, RD = recommended dose from only chemical fertilizer, Data are mean ± SE (*n* = 4). Averaged values within a column, succeeded by different small letters (a, b, c) differ significantly, ** indicates significant at 1% level of significance, *** indicates significant at 1% level of significance.

## Data Availability

The raw data supporting the conclusions of this article will be made available by the authors, without undue reservation.

## Supplementary Materials

The following are available online at https://www.mdpi.com/article/10.3390/plants10112547/s1, Table S1: List of abbreviations.
