# Supplementary material for: Integrated Nutrient Management Enhances Productivity and Nitrogen Use Efficiency of Crops in Acidic and Charland Soils
Source: Plants (Basel). 2021 Nov 22;10(11):2547. doi: 10.3390/plants10112547 (PMC8621362; doi:10.3390/plants10112547)
Supplement: Supplementary file 1 [file plants-10-02547-s001.zip › plants-1407631-supplementary.pdf]

Table S1. List of abbreviations

IPNS	:	Integrated Plant Nutrient System
PM	:	Poultry Manure
VC	:	Vermicompost
OF	:	Compost
RHB	:	Rice Husk Biochar
PMB	:	Poultry Manure Biochar
NUE	:	Nitrogen Use Efficiency
TN	:	Total Nitrogen
BD	:	Bulk Density
CD	:	Compost
OC	:	Organic Carbon
CEC	:	Cation Exchange Capacity
SOC	:	Soil Organic Carbon
AEZ	:	Agro-Ecological Zone
RCBD	:	Randomized Complete Block Design
FRG	:	Fertilizer Recommendation Guide
RD	:	Recommended Dose from Only Chemical Fertilizer
BARI	:	Bangladesh Agricultural Research Institute
BRRI	:	Bangladesh Rice Research Institute
DAT	:	Days after Transplanting
DAS	:	Days after Sowing
MWD	:	Mean Weight Diameter
GMD	:	Geometric Mean Diameter
SI	:	Stability Index
ANOVA	:	Analysis of Variance
MaAS	:	Proportional Macro Aggregate Mass
MiAS	:	Proportional Micro Aggregate Mass
WSA	:	Water-Soluble Macroaggregate
